# Comparative Protective Effects of N-Acetylcysteine, N-Acetyl Methionine, and N-Acetyl Glucosamine against Paracetamol and Phenacetin Therapeutic Doses–Induced Hepatotoxicity in Rats

**DOI:** 10.1155/2018/7603437

**Published:** 2018-09-02

**Authors:** Tahia H. Saleem, Nagwa Abo El-Maali, Mohammed H. Hassan, Nahed A. Mohamed, Nashwa A. M. Mostafa, Emaad Abdel-Kahaar, Azza S. Tammam

**Affiliations:** ^1^Department of Medical Biochemistry, Faculty of Medicine, Assiut University, Assiut, Egypt; ^2^Department of Chemistry, Faculty of Science, Assiut University, Assiut, Egypt; ^3^Department of Medical Biochemistry, Faculty of Medicine, South Valley University, Qena, Egypt; ^4^Department of Histology, Faculty of Medicine, Assiut University, Assiut, Egypt; ^5^Department of Medical Pharmacology, Faculty of Medicine, South Valley University, Qena, Egypt; ^6^Institute of Pharmacology of Natural Products & Clinical Pharmacology, Ulm University, Ulm, 89081, Germany

## Abstract

**Background and Aims:**

Both paracetamol (PA) and phenacetin (PH) are analgesic and antipyretic agents. Part of phenacetin therapeutic activity is attributed to its metabolism into paracetamol. Paracetamol causes direct hepatic oxidative stress damage. The present study aimed to investigate the possible damaging effects of both PA and PH, when used in therapeutic doses, on rat liver and to compare the antioxidant and hepatoprotective effects of N-acetylcysteine (NAC), N-acetyl-methionine (NAM), and N-acetylglucosamine (NAG) against PA- or PH-induced hepatic damage.

**Methods:**

90 male Wistar albino rats (120-140 gm) were undertaken, categorized randomly into 9 groups of 10 rats each, and administered by gavage for 2 weeks with DMSO 1% (controls), PA, PA+NAC, PA+NAM, PA+NAG, PH, PH+NAC, PH+NAM, and PH+NAG. Biochemical assays of malondialdehyde (MDA), nitric oxide (NO), reduced glutathione (GSH), total thiols, and alpha-fetoprotein (AFP) in liver homogenates and serum assays of ALT, AST, 8-hydroxy guanine (8-OH-Gua), and AFP were done. Also histopathological examinations of liver tissues in various groups were done.

**Results:**

PA and PH cause significant increase in hepatic levels of MDA, NO, and AFP and serum ALT, AST, and 8-OH-Gua levels, with significant decrease in hepatic GSH and total thiols. NAG and NAC significantly improve the PA- and PH-induced hepatic and blood, biochemical, and histopathological disturbances, respectively.

**Conclusions:**

Both PA and PH induce oxidative stress in rat liver within their therapeutic doses. NAG and NAC in pharmacological doses can antagonize the oxidative damaging effect of both PA and PH.

## 1. Introduction

Liver is frequently exposed to metabolic insults due to its major role in metabolism and detoxification of endogenous and exogenous compounds including drugs and xenobiotics [1]. Both phenacetin (mostly due to its conversion to paracetamol) and paracetamol are hepatotoxic in animals [2].

Phenacetin was extensively used as an analgesic and fever-reducing agent for many years; however, its use was prohibited in the late 1970s due to its potential to cause renal nephropathy [3]. Phenacetin was replaced by the metabolite paracetamol (acetaminophen) which is nowadays one of the most commonly used antipyretic and analgesic drugs [4], and one of the well-known experimental models of hepatotoxicity [5]. Part of phenacetin therapeutic activity is attributed to its metabolism into paracetamol [6]. Acetaminophen is converted N-acetyl-p-benzoquinone imine (NAPQI) as an intermediate toxic metabolic product via cytochrome P450, which is nullified in the liver via binding to nonprotein sulfhydryl reduced glutathione (GSH) resulting in direct hepatic oxidative stress damage due to depletion of the antioxidant capacity (glutathione peroxidase) of the liver [7, 8]. Many studies prove the antioxidant properties of N-acetyl cysteine, N-acetyl methionine, and N-acetyl glucosamine [9–11].

N-acetylcysteine (NAC) is a precursor of the amino acid L-cysteine which is a component of the biologic antioxidant glutathione (GSH). In addition to its indirect antioxidant effect (through incorporation in GSH formation), NAC exhibits also direct antioxidant properties through the interaction of its free thiol group with the electrophilic groups of ROS [12].

N-acetyl-L-methionine (NAM) is capable of replacing the dietary requirements for methionine. Methionine is an essential methyl donor in mammals and acts as an efficient scavenger of several oxidizing molecules. Methionine is also required for synthesis of cysteine which is the limiting amino acid for GSH synthesis [13].

N-acetylglucosamine, an amide of glucosamine and acetic acid, has a beneficial effect in the treatment of joint disorders, e.g., osteoarthritis and rheumatoid arthritis [14]. Previously, it was also reported that NAG has the ability to inhibit the release of superoxide anion from human polymorphonuclear leukocytes. The mechanism of this effect is not fully understood [15].

Several studies have investigated the damaging effects of paracetamol, when used in toxic doses, on the liver or the efficacy of various antioxidants to neutralize such effect, but studies regarding phenacetin could seldom be found. This is the first study aiming to carry out biochemical and histopathological assessments of possible hepatic-oxidative stress induced by paracetamol and phenacetin, when used in therapeutic doses, in male albino rats and also to compare and detect which of the three antioxidants (N-acetylcysteine, N-acetylmethionine, and N-acetylglucosamine) has the best antioxidant and hepatoprotective efficacy against the drug-induced liver injury, if any.

## 2. Materials and Methods

### 2.1. Experimental Animals

In this study, 90 male Wister albino rats, obtained from the Animal House of the Faculty of Medicine, Assiut University, Assiut, Egypt, were used. Their body weights ranged from 120 to 140 gm. Rats were housed in cages, kept at room temperature with normal 12h light/12h dark cycle, and treated according to the guidelines of the Animal House of Assiut University, where standard commercial pellets for feeding, water* ad libitum*, and other animal health conditions during the course of the experiment were performed.

### 2.2. Design of the Experiment

**Figure 1:**
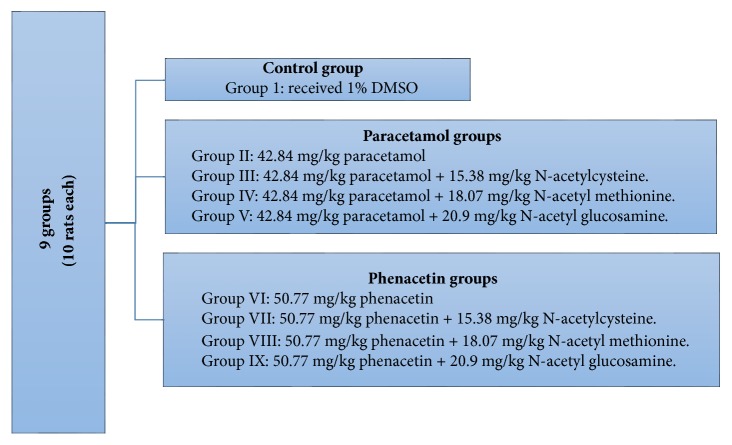
Study design: rats were divided randomly into 9 groups of 10 rats each.

The included rats were divided randomly into 9 groups of 10 rats each. All chemicals were dissolved in 1% dimethyl sulfoxide (DMSO) and rats were treated daily for 15 days. The different groups of the rats and their treatment regimens were illustrated in Figure 1.

- All used chemicals were purchased from Sigma Aldrich Chemical Co. (UK). All used reagents were of analytical grade and highest purity. The CAS numbers for PA, PH, NAC, NAM, and NAG were 103-90-2, 62-44-2, 616-91-1, 65-82-7, and 7512-17-6, respectively.
- Calculations of NAC, NAM, and NAG doses were based on theoretical chemistry from previously published work [16]. We calculated the doses of these chemicals according to their molecular weight ratio (1:1), starting with the average of therapeutic dose of paracetamol 3gm/ 24h ( in adults) with its maximum dose (4g/ 24h) [4].

### 2.3. Blood Samples Collection

Rats of the different groups were anesthetized using diethyl ether inhalation and killed by cervical dislocation 24 hours after the last dose. At time of scarifying, the blood samples were collected from the retroorbital veins into plain tubes and were centrifuged at 4,000 r.p.m for 10 min, and the separated sera were used for alanine transaminase (ALT), aspartate transaminase (AST), alfa fetoprotein (AFP), and 8-hydroxy-guanine (8-OH-Gua) measurements.

### 2.4. Preparation of Liver Samples

Rats' livers were quickly removed and washed with isotonic saline solution 0.9%, and each one was divided into two parts.

The first part was fixed immediately in 10% neutral buffered formalin for 48 hours at room temperature and then processed to prepare the paraffin sections. Serial sections of 7 *μ*m thickness were cut and subjected to haematoxylin and eosin staining (H&E) to examine the general histological changes of the liver. All these histological techniques were done in Histology Department, Faculty of Medicine, Assiut University, according to Bancroft and Gamble [17].

The second part was frozen in ice bath during scarifying and then washed, and 300 mg of the liver tissue was homogenized in 3 ml (0.1M) phosphate buffer (pH 7.4) to prepare 10% W/V homogenates, using homogenizer (Glas-Col, USA). The homogenates were centrifuged at 6,000 r.p.m for 1 hour at 4°C (MIKRO 220R Germany) and the isolated supernatants were preserved at -20°C for the subsequent biochemical measurements in the form of malondialdehyde (MDA), nitric oxide (NO), reduced GSH, total thiols, and AFP.

### 2.5. Biochemical Assays

**Figure 2:**
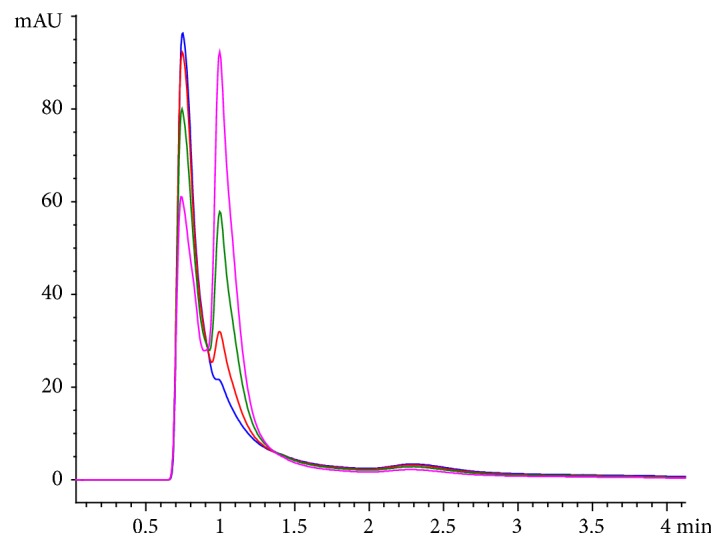
Calibration curves for HPLC assay of 8-OH-Gua at concentrations 1, 5, 15, and 25 mg/L.

(1) Serum AST and ALT measurements were done, by colorimetric method (UNICO 1200), using commercially available assay kits supplied by Egyptian Company for Biotechnology (S.A.E) with catalog no. 292002 and 291002, respectively.

(2) AFP assays in serum and hepatic tissue homogenates assays were done, using commercially available ELISA assay kits supplied by CHECK, INC (USA) with catalog no. 40-052-115007.

(3) Serum 8-OH-Gua determination was done, using high performance liquid chromatography (HPLC; Agilent Technologies 1200 Series, G1315D DAD): a 20 *μ*l serum was centrifuged at 13,000 rpm for 5 min. After centrifugation, the sample was automatically injected into the HPLC column (Zorbax Extend C18 Analytical 4.6 × 150 mm 5-um). The detector Diode Array Detector (DAD) was at 254 nm. The conditions for the HPLC were as follows: mobile phase: 100.0% (NaOAc 50 mM); pH: 4.6; flow rate: 1.5 ml/min.; column temperature: 35°C. The concentration of 8-OHG was calculated from the obtained standard curve (Figure 2).

(4) MDA, NO, reduced GSH, total thiols, and total proteins measurements in hepatic tissue homogenates were done using chemical methods (SpectraMax Plus, Molecular Devices, USA) according to Wills [18], Paya et al. [19], Beutler [20], Ellman [21], and Lowry et al. [22], respectively.

### 2.6. Statistical Analysis

Statistical Package for Social Science (SPSS) for Windows, version 5.0 (Graph Pad software, Inc., San Diego, CA, USA) was used. Experimental data were expressed as mean ± standard deviation (SD). The results were analyzed using one way analysis of variance (ANOVA) followed by Newman-Keuls multiple comparison test as a posttest to determine significant differences between means. The level of significance was considered when p<0.05.

## 3. Results

### 3.1. Biochemical Analysis Results

The mean ± SD values of serum alanine aminotransferase (ALT), aspartate aminotransferase (AST) levels, AFP, and 8-OH-Gua among the studied groups were presented in (Table 1). Phenacetin causes significant increase in the serum levels of ALT and 8-OH-Gua (127.1 U/l ± 24 and 4.643 mg/l ± 1.033, p<0.001) more than paracetamol (124.9 U/l ± 38.25 and 3.643± 1.011mg/l, p<0.01), when compared with the control group (81.45 U/l ± 37.8 and 2.240 mg/l ± 1.072), respectively. Both PA and PH cause significant increase in the AST serum levels (150.5 U/l ± 50.77 and 150.7 U/l ± 57.52, p<0.01) in comparison with the control group (100.2 U/l ± 27.84), with nonsignificant changes in serum levels of AFP (12.58 ng/ml ± 0.6175 and 12.74 ng/ml ±0.6226), when compared with the controls (12.23 ng/ml ± 0.4265, p>0.05), respectively.

Cotreatment of the rats with NAC, NAM, or NAG was associated with a significant decrease in serum levels of ALT, AST, and 8-OH-Gua in the paracetamol (73.11 U/l ± 17.08, 63.34 U/l ± 10.03, and 60.58 U/l ± 12.23; 66.93 U/l ± 32.64, 53.34 U/l ± 14.24, and 55.38 U/l ± 15.22; 0.8158 mg/l ± 0.3600, 1.981 mg/l ± 0.3059, and 1.097 mg/l ± 0.2448) and phenacetin groups (55.46 ± 17.76 U/l, 52.85 U/l ± 13.29, and 54.46 U/l ± 14.37; 76.82 U/l ± 26.64, 60.57 U/l ± 7.75, and 59.00 U/l ± 14.23; 1.535 mg/l ± 0.1212, 1.892 mg/l ± 0.7727, and 1.026 mg/l ± 0.4326), respectively, when compared with drug-only groups. The levels of significance were similar (p<0.001) among paracetamol and phenacetin groups treated with either NAC or NAG but phenacetin-NAM group showed higher significant decrease in the serum 8-OH-Gua levels (p<0.001) than in paracetamol-NAM group (p<0.01).

The mean ± SD values of hepatic homogenate levels of oxidants (MDA and NO), antioxidants (GSH and total thiols), and AFP among the studied groups are shown in (Table 2). Both paracetamol and phenacetin significantly increase the liver homogenate levels of MDA, nmol/mg tissue protein (76.20 ± 10.55 and 80.25 ± 10.5); NO, nmol/mg tissue protein (621.7 ± 51.87 and 776.5 ± 154.3); and AFP, nmol/mg tissue protein (375.9 ± 33.65, and 433.2 ± 20.03) with significant decrease in GSH, nmol/mg tissue protein (354.0 ± 61.68 and 331.3 ± 60.81), and total thiols, nmol/mg tissue protein (1299 ± 173.9 and 1215 ± 209.1), in comparison with the controls (55.78 ± 16.08, 549.0 ± 101.1, 312.2 ± 38.44, 493.9 ± 98.85, and 2062 ± 667.5, respectively).

Regarding the cotreated NAC, NAM, and NAG-paracetamol groups, there was significant reduction in the MDA (63.56 ± 9.84, 54.13 ± 13.9, and 55.50 ± 14.48), NO (566.0 ± 94.18, 520.0 ± 124.6, and 449.4 ± 68.64), and AFP (282.9 ± 26.32, 258.1 ± 65.42, and 223.9 ± 65.42), with significant increase in GSH levels (494.4 ± 94.35, 449.3 ± 111.9, and 508.8 ± 87.90), respectively, when compared with the PA-only group, except for total thiols which showed nonsignificant changes (p>0.05). These antioxidant effects were more obvious in NAG-treated group (p<0.01 for MDA, NO, and GSH; p<0.001 for AFP) than in NAC-PA and NAM-PA groups.

Regarding the cotreated phenacetin groups, the least antioxidant effect was for those received NAM as there were nonsignificant effects on homogenate levels of MDA (66.33 ± 21.16), GSH (450.0 ± 101.8), or total thiols (1615 ± 361.3), but there was significant decrease in both NO (581.5 ± 161.3, p<0.05) and AFP (277.4 ± 75.51, p<0.01), in comparison to the PH-only group. The best antioxidant effects were for those receiving NAC which cause significant decrease in MDA (58.11 ± 20.13) and NO (630.1 ± 154.5) with p<0.05 for both, AFP (300.1 ± 66.74, p<0.01), with significant increase in GSH (531.3 ± 179.5) and total thiols (1401 ± 167.4) with p<0.01 for both, when compared with the PH-only group. Those treated with NAG revealed significant decrease in MDA (53.63 ± 14.98, p<0.05), NO (495.4 ± 111.2, p<0.01), and AFP (234.7 ± 55.39, p<0.001), with significant increase in GSH (507.5 ± 77.97, p<0.05), but nonsignificant effect has been noticed on the total thiols, in comparison to the PH-only group.

### 3.2. Histopathological Examination Results of the Liver Sections

**Figure 3:**
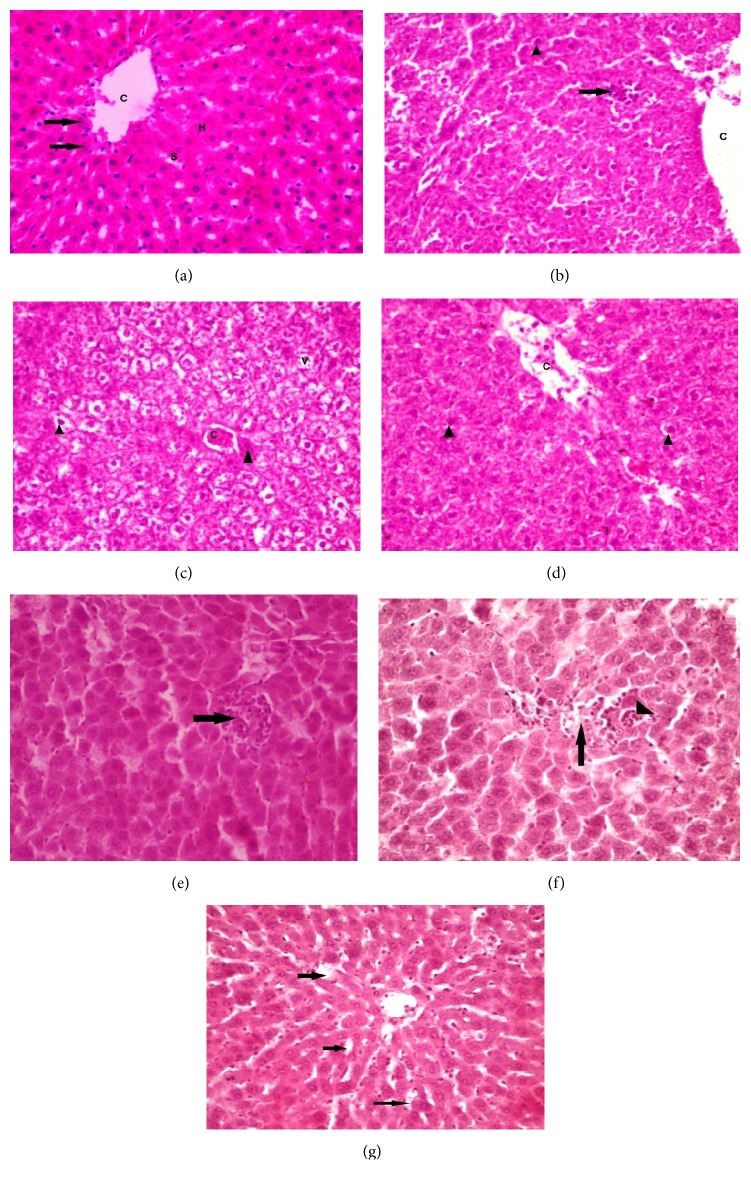
**Histological changes in the rats' liver of various paracetamol groups.** (a) Photomicrograph of a section in the liver of ***group I*** showing a normal hepatic structure. The hepatocytes are arranged in the form of plates radiating from the central vein (C). They are polyhedral with acidophilic granular cytoplasm (H). Binucleated cells are common arrows. Hepatic sinusoids appear as narrow spaces between the hepatic plates (S). (b) ***Group II*** showing dilated central vein (C) with areas of cellular infiltration in between the hepatocytes (arrow). Notice the hepatocytes with deeply stained nuclei (arrowhead). (c) ***Group II*** showing congested central vein (C). Notice many hepatocytes with vacuolated cytoplasm (V). (d) ***Group III*** showing decrease in hepatocytes vacuolation but still there are deeply stained nuclei (arrowhead) and dilated congested central veins (C) with exfoliated cells. (e) ***Group IV*** showing minimal cellular vacuolation but there is moderate cellular infiltration (Arrow). (f) ***Group V*** showing moderate cellular infiltration in between hepatocytes (arrow head) and in liver sinusoids (arrow). (g) ***Group V*** showing slight dilatation of blood sinusoids (arrow). H&E x400.

**Figure 4:**
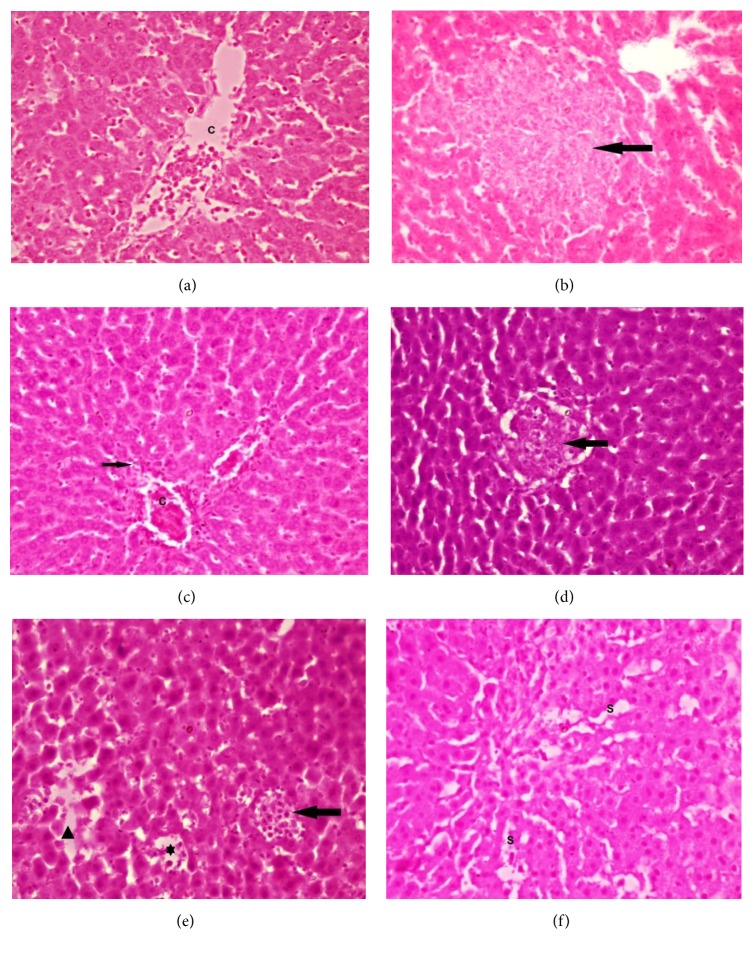
**Histological changes in the rats' liver of various phenacetin groups.** (a) Photomicrograph of a section in the liver of ***group VI*** showing dilated central vein with many cells in the lumen (C). (b) ***Group VI*** showing large irregular area of disturbed liver architecture (arrow). (c) ***Group VII*** showing minimal cellular infiltration (arrow) with dilated congested central veins (C). (d) ***Group VIII*** showing a localized area of disturbed architecture that contains many cells with vacuolated cytoplasm (arrow). (e) ***Group IX*** showing area of cellular infiltration (arrow) and the other area showing degenerated hepatocytes with many spaces (arrow head). Notice the dilated blood sinusoids with some cells in the lumen(*∗*). (f) ***Group IX*** showing dilated blood sinusoids (S). H&E x400.

Histopathological changes in the liver architecture of various study groups have been described in Figures 3 and 4. The overall results showed that both paracetamol and phenacetin cause disturbances in the liver architecture in the form of dilated congested central vein, deeply stained hepatocytic nuclei with vacuolated cytoplasm (more in paracetamol treated group), and cellular infiltrations (more in phenacetin treated group), with improvement of these changes approaching the control architecture in variable degrees among groups receiving antioxidants with the best notable improvement for PA-NAG group as regards paracetamol groups and for PH-NAC group as regards phenacetin groups, which were in line with the biochemical analysis results.

## 4. Discussion

Drug-induced hepatotoxicity is a frequent event, which is difficult to determine, due to underreporting, incomplete observation of exposure, and difficulties in diagnosis or detection [23]. We choose in our study a rat model because PA-induced hepatic damage in rats resembles human liver damage, at both biochemical and histological levels [24].

Although serum AFP is primarily used as a marker for hepatocellular carcinoma, it can be also regularly elevated in a range of nonneoplastic liver diseases such as acute liver injury with extensive necrosis [25]. AFP was found to be increased in severe acetaminophen-induced liver injury and this increase was associated with a favorable outcome [26]. In the present study, there was nonsignificant change in serum AFP in PA-only group or PH-only group when compared with the controls. This indicates that the drug doses were used within the therapeutic range and did not reach the toxic doses that cause hepatic necrosis with subsequent rise in the AFP serum levels. There was a significant rise in the serum ALT and AST levels in PA-only group and PH-only group versus the control, which confirms that both PA and PH, even when used within the therapeutic doses, still induce acute liver damage and inflammation. On the other hand, the level of AFP in liver homogenate was found to be significantly increased among PA- and PH-only-treated groups with subsequent decrease among other groups cotreated with antioxidants, which indicates the occurrence of PA and PH-induced hepatic inflammation which also was confirmed with the histopathological findings of the liver sections, which was in line with Patil et al. [27] and Abd-Elfatah et al. [28], who both reported increased AFP levels in hepatic inflammation. The hepatoprotective effects of the used drugs (NAC, NAM, and NAG) have been indicated via the associated significant decrease in the serum levels of ALT and AST and the hepatic levels of AFP, which were of more or less equal effects among the cotreated PA and PH groups.

In our experiment, the PA- and PH-induced hepatic oxidative damage was evidenced by the liver histopathological findings and the significant increased hepatic levels of MDA and NO and serum levels of 8-OH-Gua, with significant decreased hepatic GSH and total thiols among PA-only and PH-only treated groups when compared with the controls. These findings were in agreement with many studies [29–33].

Although there is considerable evidence for the safety and efficacy of NAC in management of PA-induced liver injury [34], no previous studies could be traced in literature as regards the comparison between hepatoprotective and antioxidant activity of NAC, NAM, and NAG against PA- and PH-induced liver damage when used in the therapeutic doses, as most if not all researches investigate the role of many herbal products in protecting against PA overdose or toxic doses. The present study revealed that, among the three studied antioxidants, N-acetyl glucosamine has the best hepatoprotective (based on histopathological findings) and antioxidant effect (based on biochemical analysis), in preserving the liver against PA hazardous effect, even better than the well-known NAC. While, for PH-induced liver damage, NAC showed the best antagonizing effects on biochemical and histopathological levels.

*In conclusions*, the present original study confirms that both PA and PH can induce mild degree of hepatic inflammation and oxidative stress, when used within their therapeutic doses, which was not previously investigated. NAG showed better effect than the well-known NAC, in neutralizing the hepatic oxidative stress induced by PA. PA and PH can be used safely after mixing with the pharmacological dose of NAG and NAC, respectively, and could be trialed as targeted therapeutic potential in PA or PH overdose induced hepatotoxicity.

## Figures and Tables

**Table 1:** Mean ± SD of serum alanine aminotransferase (ALT), aspartate aminotransferase (AST) levels, AFP, and 8-OH-Gua among the studied groups.

**Animal groups**	**N **	**ALT (U/L)**	**AST (U/L)**	**AFP** **(ng/ml)**	**8-OH-Gua(mg/l)**
**Controls**	10	81.45 ± 37.8	100.2 ± 27.84	12.23 ± 0.4265	2.240 ± 1.072

**Paracetamol groups**					

**PA**	10	124.9 ± 38.25^a*∗∗*^	150.5 ± 50.77^a*∗∗*^	12.58 ± 0.6175^a ns^	3.643 ± 1.011^a*∗∗*^

**PA+NAC**	10	73.11 ± 17.08^b*∗∗∗*^	66.93 ± 32.64^b*∗∗∗*^	12.45 ± 0.2716^b ns^	0.8158 ± 0.3600^a*∗*b*∗∗∗*^

**PA+NAM**	10	63.34 ± 10.03^b*∗∗∗*^	53.34 ± 14.24^b*∗∗∗*^	12.43 ± 0.4281^b ns^	1.981 ± 0.3059^b*∗∗*^

**PA+NAG**	10	60.58 ± 12.23^b*∗∗∗*^	55.38 ± 15.22^b*∗∗∗*^	12.50 ± 0.2986^b ns^	1.097 ± 0.2448^b*∗∗∗*^

**Phenacetin groups**					

**PH**	10	127.1 ± 24^a*∗∗∗*^	150.7 ± 57.52^a*∗∗*^	12.74 ± 0.6226^a ns^	4.643 ± 1.033^a*∗∗∗*^

**PH+NAC**	10	55.46 ± 17.76^b*∗∗∗*^	76.82 ± 26.64^b*∗∗∗*^	12.22 ± 0.3991^bns^	1.535 ± 0.1212^b*∗∗∗*^

**PH+NAM**	10	52.85 ± 13.29^b*∗∗∗*^	60.57 ± 7.75^b*∗∗∗*^	12.03 ± 0.6099^bns^	1.892 ± 0.7727^b*∗∗∗*^

**PH+NAG**	10	54.46 ± 14.37^b*∗∗∗*^	59.00 ± 14.23^b*∗∗∗*^	12.28 ± 0.2771^bns^	1.026 ± 0.4326^b*∗∗∗*^

*∗*p<0.05, *∗∗*p<0.01, *∗∗∗*p<0.001; ns: nonsignificant (p>0.05); a: comparison with control group, b: comparison with the drug only (PA or PH) group. PA: paracetamol; PH: phenacetin; NAC: N-acetyl cysteine; NAM: N-acetyl methionine; NAG: N-acetyl glucosamine; AFP: alpha fetoprotein; 8-OH-Gua: 8-hydroxyguanine.

**Table 2:** Mean ± SD of liver homogenate levels of oxidants (MDA and NO), antioxidants (GSH and total thiols), and AFP among the studied groups.

**Animal groups**	**N**	**MDA (nmol/mg tissue protein)**	**NO (nmol/mg tissue protein)**	**GSH (nmol/mg tissue protein)**	**Total thiols (nmol/mg tissue protein)**	**AFP (nmol/mg tissue protein)**
**Controls**	10	55.78 ± 16.08	549.0 ± 101.1	493.9 ± 98.85	2062 ± 667.5	312.2 ± 38.44

**Paracetamol groups**						

**PA **	10	76.20 ± 10.55^a*∗∗*^	621.7 ± 51.87^a*∗*^	354.0 ± 61.68^a*∗∗*^	1299 ± 173.9^a*∗∗*^	375.9 ± 33.65^a*∗*^

**PA+NAC**	10	63.56 ± 9.84^b*∗*^	566.0 ± 94.18^b*∗*^	494.4 ± 94.35^b*∗∗*^	1542 ± 376.0^b ns^	282.9 ± 26.32^b*∗∗*^

**PA+NAM**	10	54.13 ± 13.9^b*∗∗*^	520.0 ± 124.6^b*∗*^	449.3 ± 111.9^b*∗*^	1640 ± 465.4^b ns^	258.1 ± 65.42^b*∗∗*^

**PA+NAG**	10	55.50 ± 14.48^b*∗∗*^	449.4 ± 68.64^b*∗∗*^	508.8 ± 87.9^b*∗∗*^	1536 ± 369.6^b ns^	223.9 ± 65.42^b*∗∗∗*^

**Phenacetin groups**						

**PH**	10	80.25 ± 10.5^a*∗*^	776.5 ± 154.3^a*∗∗*^	331.3 ± 60.81^a*∗*^	1215 ± 209.1^a*∗∗*^	433.2 ± 20.03^a*∗∗*^

**PH+NAC**	10	58.11 ± 20.13^b*∗*^	630.1 ± 154.5^b*∗*^	531.3 ± 179.5^b*∗∗*^	1401 ± 167.4^b*∗∗*^	300.1 ± 66.74^b*∗∗*^

**PH+NAM**	10	66.33 ± 21.16^bns^	581.5 ± 161.3^b*∗*^	450.0 ± 101.8^bns^	1615 ± 361.3^b ns^	277.4 ± 75.51^b*∗∗*^

**PH+NAG**	10	53.63 ± 14.98^b*∗*^	495.4 ± 111.2^b*∗∗*^	507.5 ± 77.97^b*∗*^	1504 ± 428.5^b ns^	234.7 ± 55.39^b*∗∗∗*^

*∗*p<0.05, *∗∗*p<0.01, *∗∗∗*p<0.001; ns: nonsignificant (p>0.05); a: comparison with control group, b: comparison with the drug only (PA or PH) group. PA: paracetamol; PH: phenacetin; NAC: N-acetyl cysteine; NAM: N-acetyl methionine; NAG: N-acetyl glucosamine; MDA: malondialdehyde; NO: nitric oxide; GSH: reduced glutathione; AFP: alpha fetoprotein.

## Data Availability

The data used to support the findings of this study are included within the article.
